# Factors regarding blood donation willingness and preferences toward feedback between first-time donors and repeat donors in China: a cross-sectional survey

**DOI:** 10.3389/fmed.2025.1730038

**Published:** 2026-01-13

**Authors:** Yuxiao Wang, Lusheng Liu, Xiaohong Sun, Xinquan Lan, Lan Hua, Tianzhen Li, Nizhen Jiang, Xiaoyu Zhou, Wei Zhou

**Affiliations:** 1 Occupational Health Epidemiology Research Division, Shenzhen Prevention and Treatment Center for Occupational Diseases, Shenzhen, China; 2 Department of Occupational Health Surveillance, Shenzhen Prevention and Treatment Center for Occupational Diseases, Shenzhen, China; 3 Department of Blood Transfusion, The First Affiliated Hospital With Nanjing Medical University, Nanjing, China; 4 Health Emergency Management Department, Hunnan District Center for Disease Control and Prevention, Shenyang, China; 5 Institute of Occupational Hazard Assessment, Shenzhen Prevention and Treatment Center for Occupational Diseases, Shenzhen, China; 6 Clinical Laboratory, Jiangsu Blood Center, Nanjing, China; 7 Shenzhen Prevention and Treatment Center for Occupational Diseases, Shenzhen, China

**Keywords:** blood donation, cross-sectional study, donation policy, incentive, willingness

## Abstract

**Background:**

Identifying the factors associated with blood donation willingness and preferences for feedback enables more effective strategic recruitment planning to promote the expansion of the donor pool.

**Objective:**

To determine the difference of factors influencing willingness of donation between first-time and repeat donors.

**Methods:**

A multi-center cross-sectional study was conducted across three Chinese cities, namely, Nanjing, Zhenjiang, Danyang from December 2023 to June 2024. Participants were recruited through Jiangsu Provincial Blood Center. Data were collected via self-administered online questionnaires included demographic information, willingness to donate blood, and knowledge about blood donation. Factors influencing donation willingness were identified using multivariate logistic regression analyses.

**Results:**

A total of 1,385 participants were included in final analysis, comprising 320 first-time donors and 1,065 repeat donors. After adjustment for potential confounders, high-volume donation history was associated with greater re-donation willingness than low-volume history [adjusted odds ratio (aOR) = 4.66; 95% confidence interval (CI): 2.21–9.85; *p* < 0.001]. Donors with long-term residency also demonstrated higher interest for repeat donation compared to non-permanent residents (aOR = 2.29; 95% CI: 1.55–3.39; *p* < 0.001). Additionally, comprehensive knowledge of donation contributes to enhanced willingness for repeat participation (aOR = 8.76; 95% CI: 3.24–23.66; *p* < 0.001). Donors hold personal motivation (aOR = 2.00; 95% CI: 1.40–2.86; *p* < 0.001), incentive provision (aOR = 3.33; 95% CI: 1.32–8.41; *p* = 0.011), and positive environmental experiences (aOR = 2.51; 95% CI: 1.19–5.53; *p* = 0.016) constituted significant factors enhancing re-donation willingness. Moreover, increasing incentive value (aOR = 1.70; 95% CI: 1.06–2.73; *p* = 0.029) and offering vaccination chance (aOR = 1.66; 95% CI: 1.06–2.61; *p* = 0.028) as the preferences for feedback were associated with re-donation interest. Conversely, reduced willingness correlated with adverse event experiences (aOR = 0.49; 95% CI: 0.34–0.71; *p* < 0.001) and certificate issuance (aOR = 0.60; 95% CI: 0.42–0.84; *p* = 0.003).

**Conclusions:**

The factors influencing willingness differ significantly between first-time and repeat blood donors. Interventions should prioritize strengthening and disseminating knowledge among first-time donors to attract potential new donors. For repeat donors, enhancing the experience and implementing favorable policies are recommended to maintain donor pool. These targeted strategies would enhance donation rates in the future by governmental support.

## 1 Introduction

Voluntary blood donation, as an aspect of the social health system, is an essential prerequisite for providing blood products to clinical patients. Currently, blood donation is widely advocated to save lives and improve medical charity programs. However, health system in China is currently facing an increasingly severe blood shortage crisis due to an aging population and a decline in donors (1). Therefore, exploring the factors associated with the willingness between existing donors and potential first-time donors is important for maintaining the donor cohort and expanding the backup storage.

According to the recommendation of the World Health Organization (WHO), a whole blood donation rate of at least 1% in a country can theoretically meet clinical demands (2, 3). However, the whole blood donation rates varied substantially between regions worldwide in reality, particularly in high-income and low-income countries (4). Additionally, the gap between demand and supply is enormous, resulting in uneven distribution in many low-income countries. More than 40% global blood supply is provided by high-income countries, even though these countries account for <20% of world's population. In contrast, more than half of countries, particularly in south Asia, Oceania, and sub-Saharan Africa, lack sufficient blood supply due to the low blood donation rates (5). Although several Asian countries have reported high blood donation rates, such as 5.04% in Korea (6), the whole blood donation rate in China was 12 donations per 1,000 individuals in 2021, which barely meets the nation's essential blood demand. Moreover, the blood re-donation rate was reported only 4.8% among the previous donors in a prospective randomized controlled trial study (7). Therefore, the China blood donation program faces great challenges with the rapid growth of healthcare service requirements among aging recipient population, the loss of young donors, a declining birthrate, and emerging novel epidemics.

At present, the factors that affect blood donation have not been fully understood and may vary from country to country. To ensure a sufficient blood transfusion supply, abundant studies focusing on the factors related to willingness have been carried out (7–15). Generally, helping patients is defined as the major objective of blood donation (16–19). In contrast, self-perception of poor health, concerns over health risks or negative effects, fear, and anxiety were the main reasons for not donating (20, 21). Besides, fear of anemia, lack of opportunity or time, and profession are all related to donation practice (22). Hence, addressing the above barriers would contribute to improving the blood donation rates (23–25).

The recruitment methods, services, and incentives are all considered as breakthrough points (26). Among them, donation incentives, frequently described as extrinsically motivated encouragement intended to stimulate donation behavior, can manifest in either monetary or non-monetary forms (27). Financial incentives, such as cash, tax benefits, and gifts, are employed to improve donation program (28). In China, the prepaid cellular phone cards were found to be the most popular incentive 6 years ago (15). Many cities have also implemented their local policies to improve their blood donation programs, such as awarding extra senior high school entrance examination points for donors' children. Similarly, half-day or full-day vacation, which are preferred by blood donors, are also commonly granted to them (12). However, there are few studies focus on exploring the factors associated with maintaining re-donation among prior donors who account for a great part of blood resource (29). Even though China has a vast population of more than 1.4 billion, the blood supply still confronts a significant challenge in voluntary donation.

In the past 5 years, the blood donor pool in mainland China shrank due to Covid-19 and can't meet the increasing clinical blood demand. Existing research mainly focuses on first-time blood donation factors, but there's insufficient systematic study on repeat blood donation maintenance, feedback preferences, and a lack of dynamic analysis of the post-epidemic donor group in China. Identifying factors affecting public willingness for repeated blood donation and understanding donors' feedback expectations are crucial for formulating blood donation policies, maintaining the donor cohort, and potentially increasing donations (30). Moreover, as the concept of blood donation changes, a comprehensive analysis of re-donation factors benefits China and other developing countries. Hence, we designed a questionnaire to explore the potential factors and provide a theoretical basis for formulating strategies and guidelines to improve the blood donation program in response to China's worsening blood shortage.

## 2 Methods and materials

### 2.1 Study design

This cross-sectional survey, which originated from a registered randomized control trial, was carried out in Jiangsu province of China during the period from December 2023 to June 2024. The study has been approved by the ethics committee of the first affiliated hospital with Nanjing Medical University (No. 2021-094). The survey primarily relied on a designed questionnaire. A pilot study involving 20 participants was carried out to refine the survey instrument, and a 100% response rate was attained. The questionnaire was further revised, and its reliability and construct validity were verified.

### 2.2 Participants

The inclusion criteria for blood donors were: (a) aged 18–60 years; (b) voluntary whole blood donation; (c) weight ≥50 kg; (d) negative for HIV, hepatitis B, hepatitis C, and syphilis screening by rapid assay; (e) fully understanding the study's purpose and method, willing to participate, and signing written informed consent. The exclusion criteria were: (a) aged <18 or >60 years; (b) pregnancy; (c) using antibiotics within 48 h before blood donation, taking immunological drugs, or undergoing immunosuppression therapy in the past 6 months; (d) organ transplant receiver or blood transfusion within the last year; (e) Laboratory re-identification of infectious disease infections; (f) medical, psychological, social, or other conditions inconsistent with the protocol or affecting the ability to sign informed consent as assessed by clinician.

### 2.3 Study procedures

All potential blood donors were first screened by HIV, HBV, HCV rapid test and ABOs test at blood donation mobile health vehicles. After excluding invalid donors, eligible donors had a brief physical examination by nurses (including blood pressure, pulse rate, and body temperature measurement) and then started blood donation. According to the legal and regulatory standards in China, donors can only choose to donate whole blood in volumes of 400, 300, or 200 ml each time. For apheresis platelets, the donation amount is 1–2 therapeutic units, with each therapeutic unit not exceeding 200 ml. Prior to recruitment, donors were clearly informed of the study purpose, procedures, and potential risks/benefits. They were explicitly told that refusal to participate would not affect their blood donation services or subsequent benefits, and participation was entirely voluntary. Only those who signed the written informed consent form proceeded to the survey. Following the recruitment process, participants completed anonymous questionnaires via WeChat QR code, providing only a unique blood donation code (for data verification) instead of personal identifiers (e.g., name). This code was not linked to any personal information in the final dataset. After donation, participants stayed for 15-min safety observation and received incentives.

### 2.4 Questionnaire

In the pilot study, feedback regarding the clarity, length, and item relevance of the questionnaire was gathered. Based on this feedback, the questionnaire was revised and subsequently validated. The validation results indicated a high level of internal consistency (Cronbach's α = 0.85) and outstanding construct validity (Kaiser Meyer Olkin, KMO = 0.91). Participants in the pilot study were excluded from the subsequent investigation. This formal questionnaire had several sections: socio-demographic characteristics, health status, donation willingness and attitude, and incentive attribute preference To ensure validity, an expert panel (including an epidemiologist, blood collection nurses, blood center laboratory director, and chief transfusion technician) formulated and evaluated all items. The socio-demographic section included variables like gender, age, and educational attainment. Basic health status information covered infectious diseases, chronic conditions, and medication. Also, the survey included blood donation willingness, attitudes, and incentive preferences. The details of the questionnaire were presented in Supplementary File 1. A password-protected master identification list was stored exclusively on investigator device with limited access. Data was encrypted and regularly backed up to maintain the confidentiality and reduce data loss risks.

### 2.5 Sample size

This study aimed to investigate China's blood re-donation rate and factors influencing re-donation willingness. In China, the blood donation rates exhibit variations across different regions. Based on the donation history data from the Jiangsu Provincial Blood Center, the estimated blood donation rate of the general population ranges from 10 to 35%, and the re-donation rate stands at 60% among donors. Hence, we assumed first donors would be 35% and repeat donors would be 50%. A 95% confidence level (α = 0.05) and 90% statistical power (β = 0.10) were set. Considering a 10% dropout rate, a minimum sample size of 510 participants was needed. The sample size was calculated using Power Analysis and Sample Size software (Version 15.0.5).

### 2.6 Statistical analysis

Descriptive statistical methods were used to present background characteristics and willingness profiles. The *Kolmogorov-Smirnov* test evaluated data normality for continuous variables. Normally distributed data were described by mean ± SD and analyzed with the *t*-test, while non-normally distributed data were presented as median with IQR and analyzed by the *Mann-Whitney* tests. The *Fisher's exact* test or the chi-square test was used for categorical data like AEs. A binary logistic regression model was used to study the factors influencing vaccination willingness. Then, a multivariable logistic regression analysis was conducted by including variables with *p* ≤ 0.1 from the prior binary logistic regression analysis, while adjusting for age, gender, and education degree. Only variables with a two-tailed *p* < 0.05 were considered significant. Comparisons were shown by aORs and 95% CI. Cases with missing data were excluded. R software (version 4.3.0) was used for statistical analyses. Figures were made with GraphPad Prism (version 9.0.0), and results were presented according to the STROBE guidelines.

## 3 Results

### 3.1 Background characteristics of participants

A total of 1,460 volunteers were recruited and completed the survey. 102 questionnaires were excluded due to inconsistent responses or missing data. Finally, 1,385 questionnaires were included in analysis, with 320 (23.56%) first-time donors and 1,065 (76.44%) repeat donors. Even with the higher-than-expected proportion of repeat donors in the actual sample, this subgroup further enhanced the statistical power for between-group comparisons. The cohort had 757 males and 628 females. Among first-time donors, half were young, while middle-elderly were more common among repeat donors. Blood types A, B, and O each accounted for 29.23%−30.71%, and only 10.68% were AB. The Rh-positive type was predominant (98.53%). Most donors (75.11%) donated 400 ml of whole blood, and the rest preferred ≤200 ml or 300 ml red blood cell components. 843 (62.08%) lacked local household registration, and 1,014 (74.67%) had long-term local residency. Also, over 60% of donors had a higher–education degree. The 40-h workweek was the most reported work duration (37.04%; Table 1).

**Table 1:** The comparison of background characteristics between first-time donors and repeat donors by *Fisher's exact* or χ^2^ test.

**Item**	**First donation (320; *n*, %)**	**Repeated donation (1,038; *n*, %)**	**χ^2^ value**	***p*-Value**
**Blood ABO type**				
A	89 (27.81)	310 (29.86)	5.322	0.150
B	100 (31.25)	297 (28.61)		
O	88 (27.5)	329 (31.70)		
AB	43 (13.44)	102 (9.83)		
**Blood Rh type**				
Rh (+)	318 (99.38)	1,020 (98.27)	2.074*	0.190
Rh (–)	2 (0.62)	18 (1.73)		
**Blood donation volume, ml**				
≤200	26 (8.13)	45 (4.30)	47.011	< 0.001
300	100 (31.25)	167 (16.09)		
400	194 (60.62)	826 (79.57)		
**Gender**				
Male	185 (57.81)	572 (55.11)	0.726	0.394
Female	135 (42.19)	466 (44.89)		
**Age, years**				
18–25	160 (50.00)	233 (22.45)	107.420	<0.001
26–30	57 (17.81)	184 (17.73)		
31–40	68 (21.25)	295 (28.42)		
41–60	35 (9.69)	326 (31.41)		
**BMI**				
<18.5	21 (2.02)	11 (3.44)	12.913	0.005
18.5–25	544 (52.41)	171 (53.44)		
25–30	370 (35.65)	89 (27.81)		
≥30	103 (9.92)	49 (15.31)		
**Marital status**				
Single	207 (64.69)	410 (39.50)	62.606	<0.001
Married	101 (31.56)	559 (53.85)		
Divorced/others	12 (3.75)	69 (6.65)		
**Local household registration**				
Yes	68 (21.25)	447 (43.06)	49.439	<0.001
No	252 (78.75)	591 (56.94)		
**Long-term local residence**				
Yes	173 (54.06)	841 (81.02)	93.983	<0.001
No	147 (45.94)	197 (18.98)		
**Educational level**				
Junior school and below	44 (13.75)	135 (13.00)	0.563	0.905
High school/technical secondary school	66 (20.62)	233 (22.45)		
College/undergraduate	191 (59.69)	606 (58.38)		
Graduate student and above	19 (5.94)	64 (6.17)		
**Career**				
Unemployed	11 (3.44)	22 (2.12)	97.331	<0.001
Freelancer	104 (32.50)	299 (28.81)		
Student	91 (28.44)	110 (10.60)		
Public servant	5 (1.56)	39 (3.76)		
Health system worker	2 (0.62)	69 (6.65)		
Professional technicians	41 (12.81)	172 (16.57)		
Staff and personnel concerned	6 (1.88)	15 (1.44)		
Commercial and service personnel	22 (6.88)	116 (11.17)		
Agricultural production personnel	1 (0.31)	9 (0.87)		
Transportation personnel	6 (1.88)	58 (5.59)		
Soldier	2 (0.62)	5 (0.48)		
Retiree	1 (0.31)	26 (2.50)		
Others	28 (8.75)	98 (9.44)		
**Working pattern**				
<40(1–4 days per week)	169 (52.81)	323 (31.12)	49.906	<0.001
40 (5 days per week)	86 (26.88)	417 (40.17)		
>40(6–7 days per week)	65 (20.31)	298 (28.71)		
**Average monthly income, RMB**				
<3,000	113 (35.30)	191 (18.40)	44.158	<0.001
3,000–5,000	54 (16.88)	166 (15.99)		
5,000–9,000	99 (30.94)	427 (41.14)		
>9,000	54 (16.88)	254 (24.47)		
**Smoking**				
Yes	101 (31.56)	240 (23.12)	9.268	0.002
No	219 (68.44)	798 (76.88)		
**Alcohol consumption**				
None	169 (52.81)	503 (48.46)	1.906	0.385
1–2 times per week	143 (44.69)	504 (48.55)		
≥3 times per week	8 (2.50)	31 (2.99)		
**Had children**				
Yes	103 (32.19)	570 (54.91)	50.534	<0.001
No	217 (67.81)	468 (45.09)		
**Number of children**				
0	217 (67.81)	468 (45.09)	52.998	<0.001
1	60 (18.75)	381 (36.70)		
2	39 (12.19)	164 (15.80)		
≥3	4 (1.25)	25 (2.41)		
**Infectious diseases**				
Yes	0 (0.00)	3 (0.29)	0.927*	>0.999
No	320 (100.00)	1,035 (99.71)		
**Chronic diseases**				
None	278 (86.88)	900 (86.70)	0.944	0.967
Digestive disorders	40 (12.50)	132 (12.72)		
Respiratory disease	1 (0.31)	2 (0.19)		
Urinary system diseases	0 (0.00)	1 (0.10)		
Metabolic disease	1 (0.31)	2 (0.19)		
Rheumatic diseases	0 (0.00)	1 (0.10)		
**Regular medication for hypertension**				
Yes	2 (0.62)	1 (0.10)	3.101*	0.140
No	318 (99.38)	1,037 (99.90)		
**Vitamin consumption**				
Yes, regular	17 (5.31)	40 (3.85)	1.302	0.521
Yes, irregular	48 (15.00)	156 (15.03)		
No	255 (79.69)	842 (81.12)		

* The asterisk means the comparison was performed by Fisher's exact analysis.

### 3.2 Characteristics of the blood donation related knowledge and information

**Figure 1:**
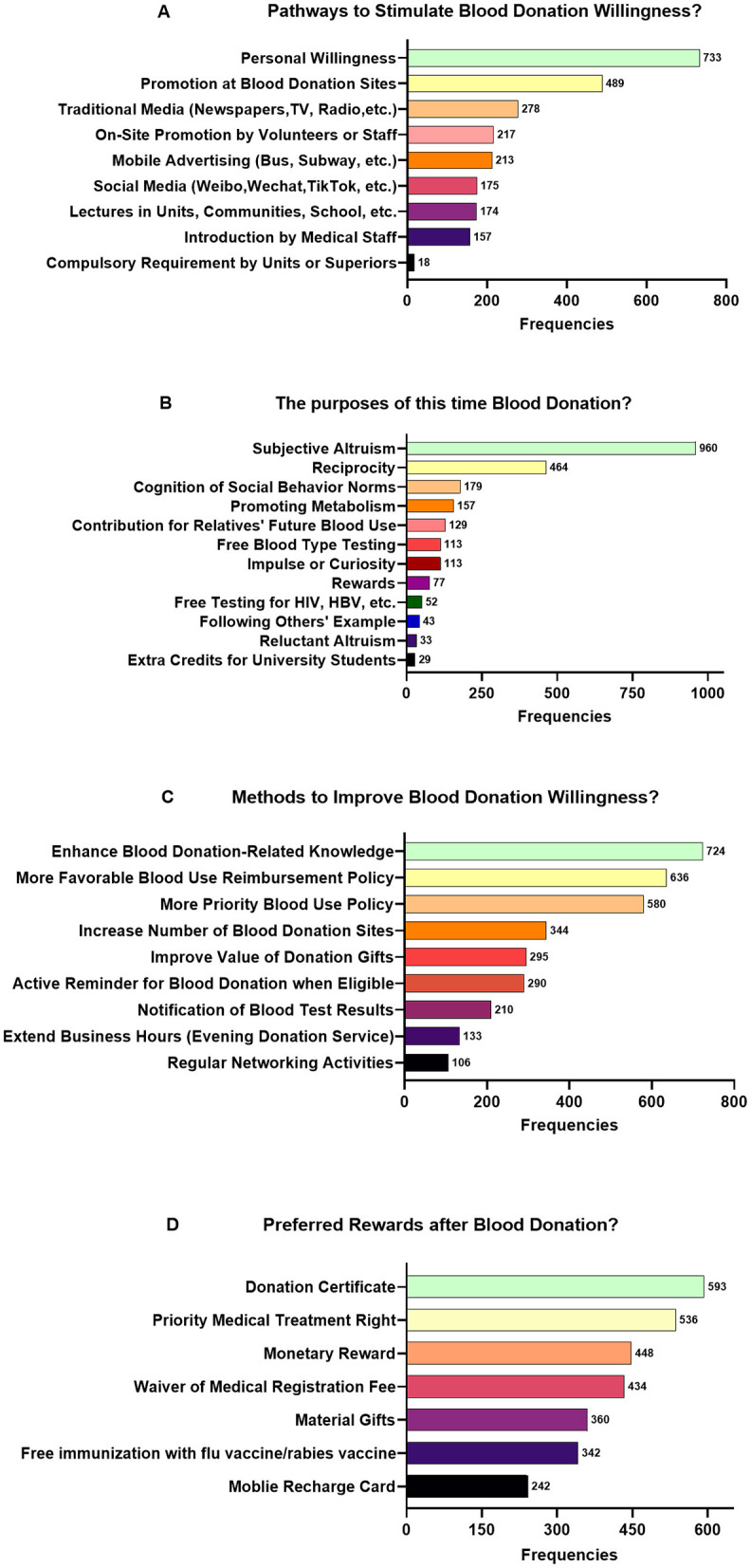
Distribution diagram of characteristics of factors associated with blood donation willingness. **(A)** The distribution diagram of the methods that stimulated donors' willingness to donate blood in this study. **(B)** The distribution diagram of the selected motivations for blood donation among donors in this study. **(C)** The distribution diagram of the methods preferred by donors for enhancing blood-donation willingness in this study. **(D)** The distribution diagram of the rewards preferred by donors after blood donation in this study.

Over half of donors were familiar with blood-donation knowledge and policies, and only 2.6% were completely unaware. Notably, first-time donors had lower awareness of donation policies than repeat donors (χ^2^ = 170.413, *p* < 0.001). For stimulating blood donation willingness this time, personal willingness was the main factor (54.0%; Figure 1A). Sub-group analysis showed that on-site promotion might have a greater influence on first-time donors, though not significantly. In contrast, repeat donors cited personal willingness at a significantly higher rate (57.6 vs. 42.2%, *p* < 0.001). Altruism was the main purpose of blood donation in both groups (Figure 1B). However, first-time donors were more likely to donate out of curiosity and for free blood-type testing (20.6 vs. 4.5% and 15.6 vs. 6.1%, respectively).

Regarding geographic preferences, 51.0% of donors prioritized city centers or commercial districts as blood-donation sites and 63.0% donors preferred 15-min commute time radius. During the blood-donation process, the technical proficiency of nurses was the key concern (461 responses, 33.9%). First-time donors expressed a higher level of anxiety regarding pain (44.7 vs. 35.8%, *p* = 0.004) and adverse reactions (44.1 vs. 32.4%, *p* < 0.001) compared to the repeat-donor group. The primary reason for non-donation across all donor groups was temporary physical unfitness (51.2%), with repeat donors reporting this reason more frequently (53.7 vs. 43.1%, *p* = 0.001). Fear of blood donation was disproportionately higher among first-time donors (26.9 vs. 11.3%, *p* < 0.001).

In terms of the donation incentives, first-time donors preferred educational initiatives related to blood-donation knowledge (59.1 vs. 51.5%, *p* = 0.018), while repeat donors prioritized policy incentives such as blood reimbursement (44.4 vs. 37.2%, *p* = 0.022; Figure 1C). Reward preferences diverged significantly: first-time donors preferred donation certificates (49.4 vs. 41.9%), whereas repeat donors favored priority medical services (41.6 vs. 32.5%, *p* = 0.004) and medical registration fee waivers (33.8 vs. 25.9%, *p* = 0.008; Figure 1D). Communication preferences also differed, with first-time donors preferring phone notifications of test results (40.0 vs. 32.6%, *p* = 0.014) and repeat donors favoring short messaging service alerts (68.6 vs. 55.9%, *p* < 0.001; Table 2).

**Table 2:** The comparison of preference profiles for incentives between first-time donors and repeat donors by *Fisher's exact* or χ^2^ test.

**Item**	**First donation (320; *n*, %)**	**Repeated donation (1,038; *n*,%)**	***X*^2^ value**	***p*-Value**
**Awareness of blood donation knowledge and policies**				
Completely unaware	23 (7.2%)	12 (1.2%)	170.413	**<0.001**
Little awareness	118 (36.9%)	119 (11.5%)		
Basic awareness	145 (45.3%)	579 (55.8%)		
High awareness	34 (10.6%)	328 (31.6%)		
**Methods to stimulate blood donation willingness?**				
Introduction by medical staff	49 (15.3%)	108 (10.4%)	5.762	**0.016**
Personal willingness	135 (42.2%)	598 (57.6%)	23.422	**<0.001**
Traditional media (newspapers, TV, radio, etc.)	60 (18.8%)	218 (21.0%)	0.762	0.383
Mobile advertising (bus, subway, etc.)	47 (14.7%)	166 (16.0%)	0.315	0.575
Social media (Weibo, WeChat, Douyin, etc.)	31 (9.7%)	144 (13.9%)	3.817	0.051
Lectures in units, communities, schools, etc.	40 (12.5%)	134 (12.9%)	0.037	0.848
Promotion at blood donation sites	127 (39.7%)	362 (34.9%)	2.459	0.117
On-site promotion by volunteers or staff	58 (18.1%)	159 (15.3%)	1.436	0.231
Compulsory requirement by units or superiors	3 (0.9%)	15 (1.4%)	0.678*	0.172
**Motivations of this blood donation?**				
Subjective altruism	201 (62.8%)	759 (73.1%)	12.546	**<0.001**
Impulse or curiosity	66 (20.6%)	47 (4.5%)	83.079	**<0.001**
Reciprocity	93 (29.1%)	371 (35.7%)	4.851	**0.028**
Free blood type testing	50 (15.6%)	63 (6.1%)	29.276	**<0.001**
Cognition of social behavior norms	32 (10.0%)	147 (14.2%)	3.702	0.054
Rewards	12 (3.8%)	65 (6.3%)	2.886	0.089
Following others' example	15 (4.7%)	28 (2.7%)	3.159	0.076
Reluctant altruism	9 (2.8%)	24 (2.3%)	0.258	0.611
Free testing for HIV, hepatitis B, etc.	14 (4.4%)	38 (3.7%)	0.339	0.561
Contribution for relatives' future blood use	35 (10.9%)	94 (9.1%)	1.007	0.316
Extra credits for university students	9 (2.8%)	20 (1.9%)	0.918	0.338
Promoting metabolism	33 (10.3%)	124 (11.9%)	0.638	0.424
**Preferred locations for blood donation sites to facilitate access?**				
Hospital or health center	128 (40.0%)	364 (35.1%)	2.576	0.109
City center or commercial districts	160 (50.0%)	532 (51.3%)	0.154	0.695
Major transportation hubs	90 (28.1%)	328 (31.6%)	1.386	0.239
Park or scenic area	110 (34.4%)	365 (35.2%)	0.067	0.796
Large shopping malls or supermarkets	95 (29.7%)	331 (31.9%)	0.550	0.458
Residential community	127 (39.7%)	456 (43.9%)	1.797	0.180
Home blood collection	42 (13.1%)	142 (13.7%)	0.064	0.800
**Preferred travel time from home/workplace to nearest blood donation site?**				
Within 15 min	196 (61.3%)	659 (63.5%)	3.724*	0.293
Within 30 min	104 (32.5%)	340 (32.8%)		
Within 60 min	14 (4.4%)	28 (2.7%)		
Within 90 min	6 (1.9%)	11 (1.1%)		
**Key concerns during blood donation process?**				
Environment	50 (15.6%)	199 (19.2%)	9.591	0.088
Blood collection nurse skills	107 (33.4%)	354 (34.1%)		
Smoothness of blood donation process	46 (14.4%)	157 (15.1%)		
Staff attitude	73 (22.8%)	190 (18.3%)		
Up-to-date blood collection equipment	15 (4.7%)	74 (7.1%)		
Others	29 (9.1%)	64 (6.2%)		
**Concerns about blood donation?**				
Pain during donation	143 (44.7%)	372 (35.8%)	8.136	**0.004**
Adverse reactions	141 (44.1%)	336 (32.4%)	14.675	**<0.001**
Risk of infectious diseases	127 (39.7%)	389 (37.5%)	0.508	0.476
Leakage of personal information	82 (25.6%)	285 (27.5%)	0.416	0.519
Impact on individual health	91 (28.4%)	290 (27.9%)	0.030	0.862
Others	46 (14.4%)	134 (12.9%)	0.457	0.499
**Main reasons for unwillingness to donate blood?**				
Fear of blood donation	86 (26.9%)	117 (11.3%)	46.839	**<0.001**
Temporarily unfit due to poor physical condition	138 (43.1%)	557 (53.7%)	10.867	**0.001**
Impact on physical function	57 (17.8%)	124 (11.9%)	7.287	**0.007**
Negative attitude from relatives and friends	25 (7.8%)	107 (10.3%)	1.736	0.188
Worry about infection	40 (12.5%)	134 (12.9%)	0.037	0.848
Low self-efficacy or concern about judgment	7 (2.2%)	24 (2.3%)	0.017	0.896
Lack of knowledge about blood donation policies	18 (5.6%)	38 (3.7%)	2.387	0.122
Time constraint	26 (8.1%)	76 (7.3%)	0.227	0.634
Unsatisfactory blood donation environment	12 (3.8%)	41 (3.9%)	0.026	0.872
Worry about illegal blood trading	41 (12.8%)	113 (10.9%)	0.903	0.342
Inconvenient blood collection	24 (7.5%)	105 (10.1%)	1.946	0.163
**Preferred methods to improve blood donation willingness?**				
Enhance blood donation-related knowledge	189 (59.1%)	535 (51.5%)	5.559	**0.018**
More priority blood use policy	119 (37.2%)	461 (44.4%)	5.218	**0.022**
More favorable blood use reimbursement policy	135 (42.2%)	501 (48.3%)	3.629	0.057
Improve value of donation gifts	58 (18.1%)	237 (22.8%)	3.187	0.074
Increase number of blood donation sites	73 (22.8%)	271 (26.1%)	1.404	0.236
Extend business hours (evening donation service)	27 (8.4%)	106 (10.2%)	0.872	0.350
Notification of blood test results	53 (16.6%)	157 (15.1%)	0.387	0.534
Active reminder for blood donation when eligible	60 (18.8%)	230 (22.2%)	1.692	0.193
Regular networking activities	21 (6.6%)	85 (8.2%)	0.899	0.343
**Preferred ways to receive test results and maintain contact after blood donation?**				
Phone notification by staff	128 (40.0%)	338 (32.6%)	6.003	**0.014**
System SMS notification	179 (55.9%)	712 (68.6%)	17.364	**<0.001**
System email notification	55 (17.2%)	143 (13.8%)	2.285	0.131
WeChat official account notification	140 (43.8%)	458 (44.1%)	0.014	0.906
Mobile app notification	54 (16.9%)	160 (15.4%)	0.393	0.531
**Preferred rewards after blood donation?**				
Donation certificate	158 (49.4%)	435 (41.9%)	5.545	**0.019**
Priority medical treatment right	104 (32.5%)	432 (41.6%)	8.512	**0.004**
Waiver of medical registration fee	83 (25.9%)	351 (33.8%)	6.980	**0.008**
Free immunization with flu vaccine/rabies vaccine	68 (21.3%)	274 (26.4%)	3.439	0.064
Monetary reward	103 (32.2%)	345 (33.2%)	0.122	0.727
Material gifts	82 (25.6%)	278 (26.8%)	0.168	0.682
Moblie recharge card	51 (15.9%)	191 (18.4%)	1.013	0.314

* The asterisk means the comparison was performed by Fisher's exact analysis. Bold values indicate statistical significance (*p* < 0.05).

### 3.3 Characteristics of the safety profiles of blood donation

Blood donation was highly safe among all donors, with the proportion of adverse reactions being merely 2.7%. The majority of adverse reactions were mild or moderate, and no serious adverse events were observed. In total, 11 (3.4%) out of 320 first-time donors and 26 (2.5%) out of 1,038 repeat donors reported at least one adverse reaction during the blood donation process (Table 3). Fever and sweating, as well as hypotension, fainting, and shock, were the two most prevalent kinds of adverse reactions among all donors (0.8 vs. 0.9%). Although the proportion of total adverse reactions reported by first-time donors was numerically higher than that of repeat donors, no statistically significant difference was detected (3.4 vs. 2.5%, *p* = 0.370). Each type of case was documented sporadically. First-time donors reported a significantly higher incidence of local pain compared to repeat donors (1.3 vs. 0.1%, *p* = 0.012). Additionally, first-time donors experienced a higher proportion of fever and sweating reactions than repeat donors (1.9 vs. 0.9%, *p* = 0.025). Conversely, repeat donors reported more cases of nausea and vomiting, facial pallor, hypotension, fainting, and shock than first-time donors, yet these differences were not statistically significant.

**Table 3:** The comparison of adverse events during the blood donation between first-time donors and repeat donors by *Fisher's exact* or χ^2^ test.

**Item**	**First donation (*n* = 320)**	**Repeat donation (*n* = 1,038)**	**χ^2^ value**	***p*-Value**
**Adverse events**				
Yes	11 (3.4)	26 (2.5)	0.803	0.370
No	309 (96.6)	1,012 (97.5)		
**Local pain**				
Yes	4 (1.3)	1 (0.1)	8.874*	0.012
No	316 (98.8)	1,037 (99.9)		
**Hematoma, bruising, and redness**				
Yes	0 (0.00)	2 (0.2)	0.617*	>0.999
No	320 (100.00)	1,036 (99.8)		
**Rash and itching**				
Yes	0 (0.00)	1 (0.1)	0.309*	>0.999
No	320 (100.00)	1,037 (99.9)		
**Nausea and vomiting**				
Yes	0 (0.00)	7 (0.7)	2.169*	0.209
No	320 (100.00)	1,031 (99.3)		
**Muscle and joint pain**				
Yes	0 (0.00)	1 (0.1)	0.309*	>0.999
No	320 (100.00)	1,037 (99.9)		
**Fever and sweating**				
Yes	6 (1.9)	5 (0.9)	5.910*	0.025
No	314 (98.1)	1,033 (99.5)		
**Facial pale**				
Yes	3 (0.9)	6 (0.6)	0.480*	0.447
No	317 (99.1)	1,032 (99.4)		
**Hypotension, fainting, shock**				
Yes	3 (0.9)	9 (0.9)	0.014*	>0.999
No	317 (99.1)	1,029 (99.1)		
**Arrhythmia, tachycardia**				
Yes	1 (0.3)	4 (0.4)	0.035*	>0.999
No	319 (99.7)	1,034 (99.6)		
**Dyspnea**				
Yes	0 (0.00)	1 (0.1)	0.309*	>0.999
No	320 (100.00)	1,037 (99.9)		
**Anxiety and irritability**				
Yes	1 (0.3)	0 (0.00)	3.246*	0.236
No	319 (99.7)	1,038 (100.00)		
**Facial and lip numbness**				
Yes	0 (0.00)	1 (0.1)	0.309*	>0.999
No	320 (100.00)	1,037 (99.9)		

* The asterisk means the comparison was performed by Fisher's exact analysis.

### 3.4 Factors associated with the willingness of blood donation between first-time donors and repeat donors

After rectifying biases by incorporating variables with *p* < 0.1 into multivariable logistic regression analysis, over 10 characteristics were identified as significant factors. Donors who previously donated 400 ml of blood were more willing to re-donate than those who donated <200 ml (aOR = 4.66, 95% CI: 2.21–9.85, *p* < 0.001). Moreover, as age advanced, 41–60 year-old donors were more likely to repeat donation than younger donors (aOR = 4.78, 95% CI: 2.03–11.23, *p* < 0.001). Additionally, repeat donors were more likely to have long-term local residency than non-permanent residents (aOR = 2.29,95% CI: 1.55–3.39, *p* < 0.001). Besides, participants who worked more than 5 days per week were more inclined to re-donate than those who worked 1–4 days per week (aOR = 2.11, 95% CI: 1.25–3.57, *p* = 0.005; Table 4).

**Table 4:** Multivariable logistic regression analysis of factors associated with the donation willingness between first-time donors and repeat donors with significant differences.

**Characteristics**	***AOR* (95% CI)**	***p*-Value**
**Blood donation volume**		
≤200 ml	Ref	
300 ml	2.06 (0.93, 4.53)	0.074
400 ml	**4.66 (2.21, 9.85)**	**<0.001**
**Age (years)**		
18–25	Ref	
26–30	1.51 (0.85, 2.67)	0.160
31–40	1.80 (0.87, 3.76)	0.115
41–60	**4.78 (2.03, 11.23)**	**<0.001**
**Long-term local residence**		
No	Ref	
Yes	**2.29 (1.55, 3.39)**	**<0.001**
**Working**		
<40(1–4 days per week)	Ref	
40 (5 days per week)	**1.80 (1.06, 3.04)**	**0.030**
>40(6–7 days per week)	**2.11 (1.25, 3.57)**	**0.005**
**Smoking**		
No	Ref	
Yes	**0.65 (0.44, 0.98)**	**0.039**
**Awareness of blood donation knowledge and policies**		
Completely unaware	Ref	
Poorly understood	1.31 (0.51, 3.40)	0.574
Basically understood	**4.03 (1.59, 10.20)**	**0.003**
Fully understood	**8.76 (3.24, 23.66)**	**<0.001**
**Introduction by medical staff**		
No	Ref	
Yes	**0.52 (0.31, 0.89)**	**0.016**
**Personal willingness**		
No	Ref	
Yes	**2.00 (1.40, 2.86)**	**<0.001**
**Impulse or curiosity**		
No	Ref	
Yes	**0.23 (0.12, 0.42)**	**<0.001**
**Free blood type testing**		
No	Ref	
Yes	**0.41 (0.24, 0.73)**	**0.002**
**Rewards**		
No	Ref	
Yes	**3.33 (1.32, 8.41)**	**0.011**
**Blood donation process**		
Others	Ref	
Environment	**2.51 (1.19, 5.33)**	**0.016**
Blood collection nurse skills	1.26 (0.64, 2.47)	0.511
Smoothness of blood donation process	1.94 (0.91, 4.17)	0.088
Staff attitude	1.18 (0.58, 2.40)	0.650
Up-to-date blood collection equipment	2.31 (0.94, 5.72)	0.069
**Adverse events**		
No	Ref	
Yes	**0.49 (0.34, 0.71)**	**<0.001**
**Feel fear toward blood donation**		
No	Ref	
Yes	**0.37 (0.23, 0.59)**	**<0.001**
**Improve value of donation gifts**		
No	Ref	
Yes	**1.70 (1.06, 2.73)**	**0.029**
**Donation certificate**		
No	Ref	
Yes	**0.60 (0.42, 0.84)**	**0.003**
**Free immunization with flu vaccine/rabies vaccine**		
No	Ref	
Yes	**1.66 (1.06, 2.61)**	**0.028**
**Local pain**		
No	Ref	
Yes	**0.01 (0.00, 0.24)**	**0.005**

Bold values indicate statistical significance (*p* < 0.05).

Notably, donors with comprehensive blood donation knowledge were more willing to repeat donation (aOR = 8.76, 95% CI: 3.24–23.66, *p* < 0.001). However, introduction by medical staffs (aOR = 0.52, 95% CI: 0.31–0.89, *p* = 0.016) and impulses or curiosity (aOR = 0.23, 95% CI: 0.12–0.42, *p* < 0.001) may decrease the re-donation rate. Furthermore, first-time donors were more likely to choose free blood type testing than repeat donors (aOR = 0.41, 95% CI: 0.24–0.73, *p* = 0.002). Nonetheless, repeat donors considered personal willingness as the key factor in enhancing willingness (aOR = 2.00, 95% CI: 1.40–2.86, *p* < 0.001). Rewards were another significant factor as increasing the re-donation willingness among existing donors (aOR = 3.33, 95% CI: 1.32–8.41, *p* = 0.011).

In the blood donation process, the environmental experience at the donation site (including air quality, lighting, and comfort) was more critical for enhancing re-donation willingness than nursing staff skills or attitudes (aOR = 2.51, 95% CI: 1.19–5.53, *p* = 0.016). Donors who had adverse events during donation, especially those with local pain or fear, were less likely to donate again (aOR = 0.49, 95% CI: 0.34–0.71, *p* < 0.001; aOR = 0.01, 95% CI: 0.00–0.24, *p* = 0.005). First-time donors with fear also refused to re-donate (aOR = 0.37, 95% CI: 0.23–0.59, *p* < 0.001). Issuing certificates post-donation had no effect on re-donation willingness (aOR = 0.60, 95% CI: 0.42–0.84, *p* = 0.003). However, increasing donation award value (aOR = 1.70, 95% CI: 1.06–2.73, *p* = 0.029) and providing a one-time flu vaccination (aOR = 1.66, 95% CI: 1.06–2.61, *p* = 0.028) attracted interest in re-donation. Variables with *p* > 0.05 were put in Supplementary Table 1.

## 4 Discussion

Blood donation contribute to saving lives, elevating the success rate of surgeries, and enhancing the quality of life. Nevertheless, the current situation in China is strikingly characterized by a low blood donation rate (31). Since a comprehend understanding of the preferences between first-time blood donors and repeat donors can significantly guide the formulation of targeted nonmonetary incentive policies, we conducted this cross-sectional study by recruited over thousands of participants. The detailed questionnaire reflected the current variations and distinctions between different population, thereby providing valuable insights into their preferences and motivations.

In this study, demographic analysis in Table 1 showed a concentration in middle-aged and elderly groups. These populations were more likely to be in the married stage and to have children than younger donors. Young donors are usually recruited from colleges, while our participants were from street donation vehicles, so the proportion of the younger age group was lower. There was a marginal male predominance. The low female blood-donation rate was mainly due to physiological factors, consistent with other studies (32). To retain female donors, more efforts should be made to encourage them to be regular donors. Repeated donors were more likely to have long-term local residency than first-time donors, indicating that long-term local residency rights are crucial for repeat donation. More than 60% of donors had tertiary education due to education popularization. As a result, highly-educated donors reported a 40-h workweek and a good financial state.

Regarding blood type distribution, A, B, O and Rh-positive were predominant, which reveals Chinese blood type characteristics and is consistent with prior study (33–35). Repeat donors are more likely to donate 400 ml than first-time donors, mainly because they know the blood donation policy and relevant knowledge and are unconcerned about adverse reactions. Also, a 400-ml donation can reduce transfusion frequency, immune reaction risk for recipients, and lower blood collection supply costs and inventory allocation pressure. Internationally, most countries adopt a 300–500 ml standard donation volume, but the proportion of 400 ml donations in China varies by city (36). To optimize blood resource utilization, efforts should be made to maintain the donation pattern of repeat donors and encourage first-time donors to donate 400 ml instead of 200 ml.

Safety of blood donation play a vital role in donation willingness (37, 38). In this study, the majority of adverse reactions were mild or moderate with no severe adverse events. Meanwhile, repeat donors demonstrated less adverse events than first-time donors, which is possibly because of their higher familiarity with donation process, stronger psychological adaptability, and gradual improvement in physical tolerance with increased donation frequency. In contrast, first-time donors tend to be more uncertain about donating due to terrible experience. Therefore, medical staff should assess first-time donors' emotions throughout donation in case of adverse effects, and pay attention to repeat donors' health status with past reaction history, enabling precise blood collection operations and risk prevention measures. Moreover, according to the China's national standard Guideline (Interventions for Donation-Related Vasovagal Reactions), consuming 400–500 ml of water 20 min before donation to reduce the risk of vasovagal reaction was recommended (39). This reaction was one of the typical adverse reactions, whereas the actual water-consuming behavior was not collected. Hence, the future research may incorporate this indicator to explore its potential associations with re-donation willingness.

In this study, general participants were familiar with blood donation knowledge and policies, with only 2.6% reporting complete unawareness. Significantly, first-time donors had lower awareness of donation policies than repeat donors. This is because first-time donors were more likely to be attracted by impulse, curiosity or free blood-type testing, while repeat donors continuously gained knowledge during re-donation. Half of donors preferred city centers or commercial districts as donation sites, and 63.0% chose sites with a commute time of within 15 min from home or workplace. Although convenience is crucial for promoting blood donation, there's no significant behavioral difference between repeat and first-time donors.

Comprehensive understanding of blood donation preferences can help procure an adequate and sustainable blood supply (40). For this blood donation instance, most donors said personal willingness was the main motivator, and sub-group analysis showed repeat donors had a significantly higher rate than first-time donors. So, efforts should be made among first-time donors by highlighting the idea of helping patients to boost personal willingness, along with promotion at blood donation sites (41, 42). Subjective altruism and reciprocity were the top two purposes of blood donation and more common in repeat donors (43). In contrast, first-time donors were more likely to donate due to impulse, curiosity, and free blood type testing, which were caused by multiple reasons. Socially, the lack of continuous public education on the long-term impact of blood donation may lead to short-term participation for immediate incentives instead of long-term altruism (44). Institutionally, poor feedback mechanisms can't strengthen the sense of contribution among first-time donors, decreasing their chance of coming back. Therefore, carrying out structured educational campaigns emphasizing the real benefits of blood donation can enhance intrinsic motivation (45–47).

In the process of blood donation, first-time donors place greater emphasis on the attitude of staff, whereas repeat donors exhibit more concerns about the environment. Besides, first-time donors exhibited a significantly higher level of anxiety regarding pain and adverse reactions compared to the repeat donor group. Therefore, enhancing the donation experience by improving service quality at donation site, including shorter wait times, comfortable facilities, and personalized post-donation follow-ups can mitigate anxiety and foster loyalty. When donors decline to donate blood, repeat donors are more likely than first-time donors to cite the temporary self-perception of poor health as the reason. Since the stringent donation screening criteria may led to a contraction in the pool of eligible donors, more frequent communication with previous donors who are temporarily physically disabled is the key approach for maintaining donation rate (48). Anxiety and fear related to blood donation are significantly more common among first-time donors. Consequently, more knowledge-based promotion is needed for this population to alleviate their fears.

Figure 1C showed the three most regarded strategies for elevating blood donation rate, such as enhancing donation-related knowledge, blood utilization priority policy, and reimbursement policy. Specifically, first-time donors preferred educational programs on blood donation knowledge (49), whereas repeat donors focused more on policy-based incentives like blood reimbursement. Additionally, improving the value of donation gifts was another preferred approach for repeat donors. Targeting different populations and making appropriate choices can help formulate more suitable and feasible policies, effectively promoting donor pool expansion and blood storage (50). First-time donors preferred certificates as rewards, while repeat donors favored priority medical services, medical registration fee exemptions, and free vaccination. According to a prior study (15) in China, current incentives are prepaid cellular phone cards, transportation cards, or movie tickets worth over $4, along with snacks like bread or milk. It is challenging to ascertain whether an incentive with monetary value can be classified as a monetary incentive. Drawbacks of this method were expected, for example, some people would delay their donation and/or only donate until there were attractive incentives. Certain experts have levied critiques against this system, positing that monetary incentives contravene the principle of the non-remunerated donation mechanism (51). Empirical evidence indicates that monetary incentives may crowd out intrinsic motivations, thus weakening donation behaviors. Although providing incentives is paradoxical in the non-remunerated blood donation initiative, it can effectively solve the problem of low blood donation rates (52).

Moreover, several donors are curious and question details on donated blood use, like hospital blood supply, application procedures, self-information safety, and blood usage priority. Non-transparency of blood usage was criticized, and the possibility of illegal use or transaction of blood products was questioned. To respond, city blood centers should let donors self-check donated blood usage on the official website. Besides, establishing a comprehensive feedback system, such as regular updates on blood utilization and recognition programs for repeat donors, can cultivate a sense of reciprocal responsibility and promote sustainable donation behavior.

The present study offers several advantages. Firstly, this cross-sectional survey gathered comprehensive data which enabled a thorough understanding of the disparities between first-time and repeat blood donors. Secondly, as this study conducted at the donation site, it achieved a high response rate. The sample size was relatively large and more likely to be representative of the broader population, which enhanced the statistical power and improved the reliability of results. Thirdly, this study tries to tackle the practical problem of enhancing blood donation willingness and sustaining donor cohorts in China. By identifying the specific factors differentiating first-time and repeat donors, the research provides actionable insights for formulating targeted interventions.

### 4.1 Limitations

Nevertheless, this cross-sectional cohort study has several limitations. Gender and age disparities between the two groups may bias result interpretations, so we controlled confounding variables in the multivariate logistic model. The single-time-point study design makes it difficult to establish causal relationships between investigated factors and repeat donation behavior due to unmeasured variables or temporal variations. Also, all participants being donors may introduce selection bias as those with positive experiences are more likely to participate, distorting results and limiting generalizability. Moreover, the fixed order of options in multiple-choice questions and excessive questions may make blood donors impatient and averse, increasing the likelihood of choosing early options. The data rely on self-reported information from donors, which may have biases like recall or social desirability bias, affecting data accuracy on motivators and concerns.

Aside from the aforementioned limitations, this study also has several drawbacks that warrant further addressing. Given that our data collection was restricted to the period from December to June, the observation window was comparatively limited. This resulted in insufficient sample sizes across potential seasonal subgroups, precluding robust statistical inference. Future studies should therefore extend data collection to a full calendar year to enable rigorous exploration of seasonal factors influencing donation behavior. Additionally, the omission of crowd pressure leads to an incomplete representation of the determinants of donation behavior. Future research should incorporate a more comprehensive scale to quantitatively analyze its association with re-donation willingness. Furthermore, since many religious teachings emphasize altruism, which may correlate with blood donation willingness, future studies should include religion as an additional potential factor to examine its association with re-donation willingness in the Chinese context.

### 4.2 Conclusion

The findings of our research indicated that the factors associated with the willingness to donate blood vary between first-time donation and repeat donation. The individual willingness, local residence and donation knowledge popularization were significantly correlated with the repeated donation behavior. Additionally, providing rewards, enhancing the value of the gifts and offering free one-time vaccine immunization also had a great impact. This suggests that repeated donation is highly dependent on the cohort formed by feedback. The optimization of national blood transfusion services and the implementation of evidence-based targeted strategies by increased government financial investment to elevate the donation rates, including augmenting knowledge dissemination among first-time donors and reinforcing policy incentives for repeat donors, are crucial for ensuring blood availability.

## Data Availability

The raw data supporting the conclusions of this article will be made available by the authors, without undue reservation.
