# Supplementary material for: Factors regarding blood donation willingness and preferences toward feedback between first-time donors and repeat donors in China: a cross-sectional survey
Source: Front Med (Lausanne). 2026 Jan 13;12:1730038. doi: 10.3389/fmed.2025.1730038 (PMC12834724; doi:10.3389/fmed.2025.1730038)
Supplement: Supplementary file 1 [file Table_1.docx]

Supplementary Table 1. Multivariable logistic regression analysis of factors associated with the donation willingness between first-time donors and repeat donors without significant differences.

| Characteristics | OR (95%CI) | *P* value |
| --- | --- | --- |
| Sex |  |  |
| Female | ref |  |
| Male | 0.81 (0.54, 1.22) | 0.315 |
| BMI |  |  |
| Normal Weight | ref |  |
| Underweight | 1.41 (0.50, 4.00) | 0.513 |
| Overweight | 0.92 (0.62, 1.37) | 0.680 |
| Obesity | 0.74 (0.43, 1.25) | 0.259 |
| Marital status |  |  |
| Single | ref |  |
| Married | 1.19 (0.52, 2.73) | 0.673 |
| Divorced/others | 1.58 (0.53, 4.75) | 0.414 |
| Local household registration |  |  |
| No | ref |  |
| Yes | 1.37 (0.88, 2.11) | 0.161 |
| Career |  |  |
| Unemployed | ref |  |
| Freelancer | 0.77 (0.19, 3.03) | 0.705 |
| Student | 0.46 (0.15, 1.41) | 0.174 |
| Public servant | 1.27 (0.19, 8.50) | 0.804 |
| Health system worker | 4.33 (0.55, 34.00) | 0.163 |
| Professional technicians | 0.52 (0.12, 2.30) | 0.390 |
| Staff and personnel concerned | 0.42 (0.07, 2.76) | 0.370 |
| Commercial and Service Personnel | 0.97 (0.22, 4.31) | 0.968 |
| Agricultural production personnel | 1.20 (0.08, 17.18) | 0.896 |
| Transportation personnel | 1.41 (0.24, 8.19) | 0.703 |
| Soldier | 1.32 (0.08, 20.57) | 0.843 |
| Retiree | 5.37 (0.42, 68.22) | 0.195 |
| Others | 0.65 (0.15, 2.87) | 0.574 |
| Average monthly income (RMB) |  |  |
| < 3000 | ref |  |
| 3000-5000 | 0.42 (0.16, 1.13) | 0.086 |
| 5000-9000 | 0.62 (0.24, 1.63) | 0.337 |
| > 9000 | 0.54 (0.20, 1.46) | 0.225 |
| Had children |  |  |
| No | ref |  |
| Yes | 0.51 (0.12, 2.20) | 0.365 |
| Number of children |  |  |
| 0 | ref |  |
| 1 | 0.92 (0.26, 3.27) | 0.896 |
| >=2 | 0.83 (0.22, 3.06) | 0.777 |
| Social Media (Weibo, WeChat, Douyin, etc.) |  |  |
| No | ref |  |
| Yes | 1.61 (0.91, 2.85) | 0.104 |
| Subjective Altruism |  |  |
| No | ref |  |
| Yes | 1.18 (0.80, 1.73) | 0.399 |
| Reciprocity |  |  |
| No | ref |  |
| Yes | 1.13 (0.77, 1.67) | 0.525 |
| Cognition of Social Behavior Norms |  |  |
| No | ref |  |
| Yes | 1.07 (0.62, 1.85) | 0.812 |
| Following Others' Example |  |  |
| No | ref |  |
| Yes | 1.09 (0.46, 2.55) | 0.847 |
| Temporarily Unfit due to Poor Physical Condition |  |  |
| No | ref |  |
| Yes | 1.25 (0.87, 1.79) | 0.230 |
| Impact on Physical Function |  |  |
| No | ref |  |
| Yes | 0.69 (0.42, 1.13) | 0.138 |
| Enhance Blood Donation-Related Knowledge |  |  |
| No | ref |  |
| Yes | 0.74 (0.52, 1.07) | 0.109 |
| More Priority Blood Use Policy |  |  |
| No | ref |  |
| Yes | 1.08 (0.71, 1.65) | 0.724 |
| More Favorable Blood Use Reimbursement Policy |  |  |
| No | ref |  |
| Yes | 0.93 (0.62, 1.41) | 0.742 |
| Phone Notification by Staff |  |  |
| No | ref |  |
| Yes | 0.80 (0.55, 1.15) | 0.222 |
| System SMS Notification |  |  |
| No | ref |  |
| Yes | 1.25 (0.86, 1.81) | 0.240 |
| Priority Medical Treatment Right |  |  |
| No | ref |  |
| Yes | 1.40 (0.93, 2.11) | 0.104 |
| Waiver of Medical Registration Fee |  |  |
| No | ref |  |
| Yes | 1.24 (0.80, 1.91) | 0.332 |
| Fever and sweating |  |  |
| No | ref |  |
| Yes | 1.08 (0.11, 10.33) | 0.949 |
