# Supplementary material for: Factors regarding blood donation willingness and preferences toward feedback between first-time donors and repeat donors in China: a cross-sectional survey
Source: Front Med (Lausanne). 2026 Jan 13;12:1730038. doi: 10.3389/fmed.2025.1730038 (PMC12834724; doi:10.3389/fmed.2025.1730038)
Supplement: Supplementary file 2 [file Table_2.docx]

**Supplementary file: The Blood Donation Willingness Questionnaire**

**1. Sociodemographic Information**

A1. **Date of Birth:** _______ Year ______ Month

A2. **Gender:** ( ) A. Male; B. Female

A3. **Marital Status:** ( ) A. Unmarried; B. Married and living together; C. Married and living separately; D. Divorced; E. Widowed; F. Other

A4. **Do you have a household registration in this city?** ( ) A. Yes; B. No

A5. **Do you reside in this city long-term?** ( ) A. Yes; B. No

A6. **Educational Background:** ( ) A. Junior high school or below; B. Senior high school/vocational high school/technical secondary school; C. College diploma; D. Bachelor's degree; E. Master's degree or above

A7. **Occupation Category:** ( )A. Unemployed; B. Freelancer; C. Student; D. Staff of state organs, mass organizations, enterprises, and public institutions; E. Medical and health professionals (hospitals/CDCs/health clinics, etc.);F. Professional and technical personnel (scientific research/engineering/education/computer/finance/culture, etc.);G. Clerical and related personnel (clerical/safety and fire-fighting personnel);H. Commercial and service industry personnel (catering/sports/beauty/automotive/real estate/wholesale/training/maintenance, etc.);I. Agricultural, forestry, animal husbandry, fishery, and water conservancy production personnel;J. Production and transportation-related personnel (food/tobacco/textile/chemical/medical manufacturing/transportation/postal storage, etc.);K. Military personnel; L. Retired; M. Other (not mentioned above)

A8. **Weekly Working Hours**: ( ) A. 1-4 days/week; B. 5 days/week (two days off); C. 6 days/week (one day off); D. 7 days/week (no days off); E. Flexible working hours

A9. **Average Monthly Income (RMB)**: ( ) A. 0-2999 yuan; B. 3000-4999 yuan; C. 5000-6999 yuan; D. 7000-8999 yuan; E. 9000-10999 yuan; F. 11000 yuan or above

A10. **Do you smoke?** ( ) A. Yes; B. No

A11. **Daily Smoking Amount:** ( ) A. One pack or more per day; B. One pack every 2-3 days; C. One pack per week; D. Occasional smoking; E. Do not smoke

A12. **Alcohol Consumption Frequency:** ( ) A. Drink every day (heavy consumption); B. Drink every day (light consumption); C. 2-3 times a week; D. Once a week; E. Occasional drinking (1-2 times a month); F. Do not drink

A13. **Do you have children?** ( ) A. Yes; B. No

A14. **Please fill in your blood type** (): A. Type A; B. Type B; C. Type A; D. Type O

A15. **Please select the Rh type of your blood** (): A.Rh positive ; B.Rh negative

A16. **What is your blood donation volume this time?** A. 200ml whole blood; B. 300ml whole blood; C. 400ml whole blood; D. Others (apheresis platelets/plasma, etc.)

**2. Personal Health and Chronic Disease Information**

B1. **Do you have any of the following infectious diseases?** ( ) A. Sexually transmitted diseases, AIDS; B. Leprosy; C. Hepatitis B surface antigen positive; D. Hepatitis C antibody positive; E. Tuberculosis; F. Influenza virus infection; G. Other infectious diseases; H. No infectious diseases

B2. **Do you have any of the following chronic diseases?** ( ) A. Digestive system diseases (chronic gastritis, peptic ulcer, intestinal tuberculosis, chronic enteritis, chronic diarrhea, chronic hepatitis, liver cirrhosis, chronic cholecystitis, etc.); B. Respiratory system diseases (asthma, chronic respiratory failure, silicosis, pulmonary fibrosis, etc.); C. Circulatory system diseases (chronic heart failure, coronary heart disease, congenital heart disease, hypertension, etc.); D. Hematological system (chronic anemia, chronic myeloid leukemia, chronic lymphocytic leukemia, etc.); E. Metabolic and nutritional diseases (hyperlipidemia, diabetes and its complications, gout, etc.); F. Rheumatic diseases (systemic lupus erythematosus, rheumatoid arthritis, etc.); G. Hematological tumors or solid tumors; H. Urinary system (chronic nephritis, chronic renal failure, chronic urinary system inflammation); I. None of the above diseases

B3. **Do you take hypertension medication regularly?** ( ) A. Yes; B. No

B4. **Do you take vitamins regularly?** ( ) A. Yes; B. No

B5. **Have you been infected with COVID-19 in the past year?** ( ) A. Yes;Infection date: ____ Year __ Month B. No

B6. **Had you donated blood before being infected with COVID-19?** ( ) A. Yes; B. No

B7. **Have you donated blood after recovering from COVID-19?** ( ) A. Yes; B. No

B8. **How many times have you donated blood in total** (including this time)? ____

B9. **Have you experienced any adverse reactions during blood donation?** ( ) A. Yes; B. No

B10. **Types of adverse reactions?** ( )A. Local pain; B. Local induration; C. Local hematoma (ecchymosis), flushing; D. Local rash, itching; E. Acute allergic reaction; F. Nausea, vomiting; G. Muscle and joint pain, headache, chest pain, back pain, abdominal pain; H. Chills; I. Fever, sweating; J. Pallor; K. Hypotension, syncope, shock; L. Spasms, convulsions; M. Generalized bleeding; N. Hemoglobinuria, oliguria, anuria; O. Urticaria; P. Dyspnea; Q. Arrhythmia, tachycardia; R. Anxiety, irritability; S. Incontinence of urine and feces; T. Numbness of the face and lips

**3. Blood Donation Willingness Information**

D1. **Which of the following channels inspired your desire to donate blood this time?** ( )A. News or media advertisements; B. Company/community promotion; C. Promotion at blood donation sites (blood collection vehicles); D. Introduction by medical staff; E. Recommendation by relatives and friends; F. Personal willingness; G. Mandatory requirement by the company or superior; H. Other

D2. **What are the main purposes of your blood donation this time?** ( )A. Subjective altruism (voluntarily increasing welfare for others); B. Reluctant altruism (being forced to follow others' wishes/involuntary); C. Curiosity (feeling novel or interesting to try); D. Incentive-based (company leave/allowance/blood donation gifts); E. Reciprocity (believing that helping others will lead to help from others in the future); F. Recognition of social norms (perceiving social pressure); G. Free blood type testing; H. Free testing for HIV/hepatitis B and other infectious diseases; I. Contributing to future blood use for relatives; J. Other

D3. **Which locations would you prefer blood donation sites to be set up for your convenience?** ( )A. Hospitals/health centers; B. City center/busy commercial areas; C. Large transportation hubs; D. Parks/scenic areas; E. Large shopping malls/supermarkets; F. Residential communities; G. Home blood collection; H. Other

D4. **Which link of the blood donation process do you care most about?** ( )A. Blood donation environment (lighting/temperature/odor/decoration/music, etc.); B. Blood collection nurses' operational skills; C. Smoothness of the blood donation process; D. Attitude of staff; E. Update of blood collection equipment; F. Other

D5. **What are your concerns about blood donation?** ( )A. Pain during blood donation; B. Risk of infectious diseases from blood donation; C. Leakage of personal information during blood donation; D. Adverse reactions ; E. Impact of blood donation on individuals; F. Other

D6. **What are the main reasons for your decision not to donate blood?** ( )A. Fear of blood donation; B. Health status not suitable for blood donation (e.g., insufficient sleep/not eating, etc.); C. Failure to meet blood donation criteria (e.g., infected with infectious diseases); D. Worry about the impact of blood donation on physical function; E. Worry about being infected with viruses or bacteria during the blood donation process; F. Low self-efficacy/worry about others' opinions/shyness; G. Lack of understanding of blood donation policies/knowledge; H. Time constraints (long blood donation time); I. Poor blood donation environment (unwilling to donate blood on mobile blood collection vehicles); J. Worry about illegal blood trading; K. Inconvenient blood collection (far from blood donation sites or short opening hours); L. Restrictions on activities after blood donation; M. Negative attitudes of relatives and friends around (discouraged by family members or friends); N. Other

D7. **What services would you like to receive after blood donation?** ( )A. More knowledge about blood donation; B. More preferential blood transfusion reimbursement policies; C. Higher priority blood transfusion policies; D. More blood donation sites; E. Higher value of gifts; F. Extended business hours (providing evening blood donation services); G. Cancel actual subsidies and realize voluntary blood donation; H. Notification of blood test results; I. Other

D8. **What kind of reward would you prefer to receive after blood donation?** ( )A. Money (red envelope); B. Items (milk, food, etc.); C. Phone recharge card; D. Notification of blood test results; E. Issuance of blood donation certificate of honor; F. Priority medical consultation right once; G. Free vaccination opportunity against seasonal influenza/tetanus/rabies once; H. Other

D9. **How well do you understand blood donation knowledge and relevant policies?** () A.High Awareness [Possesses comprehensive, in-depth knowledge of blood donation, including detailed policies, specialized information, and broader implications]; B.Basic Awareness [Masters the core, practical knowledge and key policies necessary for blood donation]; C. Little Awareness [Has a vague or minimal understanding of blood donation, limited to superficial, non-core information].; D.Completely Unaware [No knowledge of blood donation-related knowledge, policies, or procedures.]

D10. **How long would you prefer the travel time from your home/workplace to the nearest blood donation site to be?** ( ) A. Within 15 minutes; B.Within 30 minutes; C. Within 60 minutes; D.Within 90 minutes

D13. **After donating blood, through which forms would you be willing to obtain test results and maintain long-term contact?** ( ) A. Staff phone call notification; B. System SMS notification; C. System email notification; D. WeChat official account notification; E. Professional blood donation management mobile APP notification ; F. Other (please specify)
